# Natural and engineered chemokine (C-X-C motif) receptor 4 agonists prevent acute respiratory distress syndrome after lung ischemia–reperfusion injury and hemorrhage

**DOI:** 10.1038/s41598-020-68425-0

**Published:** 2020-07-09

**Authors:** Favin S. Babu, Xiaomei Liang, Garrett A. Enten, Anthony J. DeSantis, Brian F. Volkman, Xianlong Gao, Matthias Majetschak

**Affiliations:** 1 Department of Surgery, Burn and Shock Trauma Research Institute, Loyola University Chicago, Maywood, IL USA; 2 Department of Surgery, Morsani College of Medicine, University of South Florida, Tampa, FL USA; 3 Department of Biochemistry, Medical College of Wisconsin, Milwaukee, WI USA; 4 Department of Molecular Pharmacology and Physiology, Morsani College of Medicine, University of South Florida, 12901 Bruce B. Downs Blvd., MDC 3127, Tampa, FL 33612 USA

**Keywords:** Drug discovery, Physiology, Diseases, Medical research, Molecular medicine

## Abstract

We compared therapeutic properties of natural and engineered chemokine (C-X-C motif) receptor 4 (CXCR4) agonists in a rat acute respiratory distress syndrome (ARDS) model utilizing the PaO_2_/FiO_2_-ratio as a clinically relevant primary outcome criterion. Ventilated rats underwent unilateral lung ischemia from t = 0–70 min plus hemorrhage to a mean arterial blood pressure (MAP) of 30 mmHg from t = 40–70 min, followed by reperfusion/fluid resuscitation until t = 300 min. Natural CXCR4 agonists (CXCL12, ubiquitin) and engineered CXCL12 variants (CXCL12_1_, CXCL2_2_, CXCL12K27A/R41A/R47A, CXCL12 (3–68)) were administered within 5 min of fluid resuscitation. Animals treated with vehicle or CXCL12 (3–68) reached criteria for mild and moderate ARDS between t = 90–120 min and t = 120–180 min, respectively, and remained in moderate ARDS until t = 300 min. Ubiquitin, CXCL12, CXCL12_1_ and CXCL12_2_ prevented ARDS development. Potencies of CXCL12/CXCL12_1_/CXCL12_2_ were higher than the potency of ubiquitin. CXCL12K27A/R41A/R47A was inefficacious. CXCL12_1_ > CXCL12 stabilized MAP and reduced fluid requirements. CXCR4 agonists at doses that preserved lung function reduced histological injury of the post-ischemic lung and reduced mortality from 55 to 9%. Our findings suggest that CXCR4 protein agonists prevent development of ARDS and reduce mortality in a rat model, and that development of new engineered protein therapeutics with improved pharmacological properties for ARDS is possible.

## Introduction

Acute respiratory distress syndrome (ARDS) remains a major contributor to morbidity and mortality in trauma patients. Although the incidence of ARDS in trauma patients has decreased in the last decade, mortality from ARDS has increased^1^. The treatment of ARDS is currently limited to supportive therapy and lung-protective ventilation strategies. Drugs that attenuate development and progression of ARDS are not available, but highly desirable for their potential to improve outcomes.

Several lines of evidence suggest that natural and synthetic agonists of chemokine (C-X-C motif) receptor 4 (CXCR4) protect lung endothelial barrier function and have lung protective properties in animal models^2–11^. We showed previously that treatment with the non-cognate CXCR4 agonist ubiquitin protects lung endothelial barrier function during resuscitation from hemorrhagic shock and prevents development of ARDS in pre-clinical lung ischemia–reperfusion injury, endotoxemia and polytrauma models^2,5,6,10^. The therapeutic potential of the cognate CXCR4 agonist chemokine (C-X-C motif) ligand 12 (CXCL12, stromal cell-derived factor 1α) during development of ARDS, however, is unknown. Furthermore, we recently reported that various engineered CXCL12 variants, which possess biochemical and pharmacological characteristics distinct from wild-type CXCL12, protect human lung endothelial barrier function in vitro similar to wild-type CXCL12^9^. These engineered CXCL12 variants have not been tested in an in vivo lung injury model.

The aim of the present study was to test and compare efficacy and potency of ubiquitin, CXCL12 and engineered CXCL12 variants to prevent ARDS development in a rat model.

Thoracic injury and hypotension on admission have been identified as important risk factors for the development of trauma-induced ARDS in patients^1,12–14^. Thus, in the present study, we employed a rat model that mimics clinical predictors of trauma-induced ARDS development by combining a thoracic injury component, i.e. thoracotomy and subsequent lung ischemia–reperfusion injury, with systemic hypotension during the hemorrhagic shock period. This animal model was designed to suffice key criteria of the Berlin definition of ARDS^15^, i.e. to result in a ratio of arterial oxygen partial pressure to fractional inspired oxygen (P/F) < 300 mmHg under positive end-expiratory pressure ≥ 5 cmH_2_O. Our findings show that treatment with CXCR4 agonists during the early fluid resuscitation period prevents development of lung ischemia–reperfusion injury and hemorrhage induced ARDS in rats and suggest that the development of engineered CXCR4 agonists with improved therapeutic efficacy is possible.

## Results

### Ubiquitin treatment prevents development of ARDS

**Figure 1:**
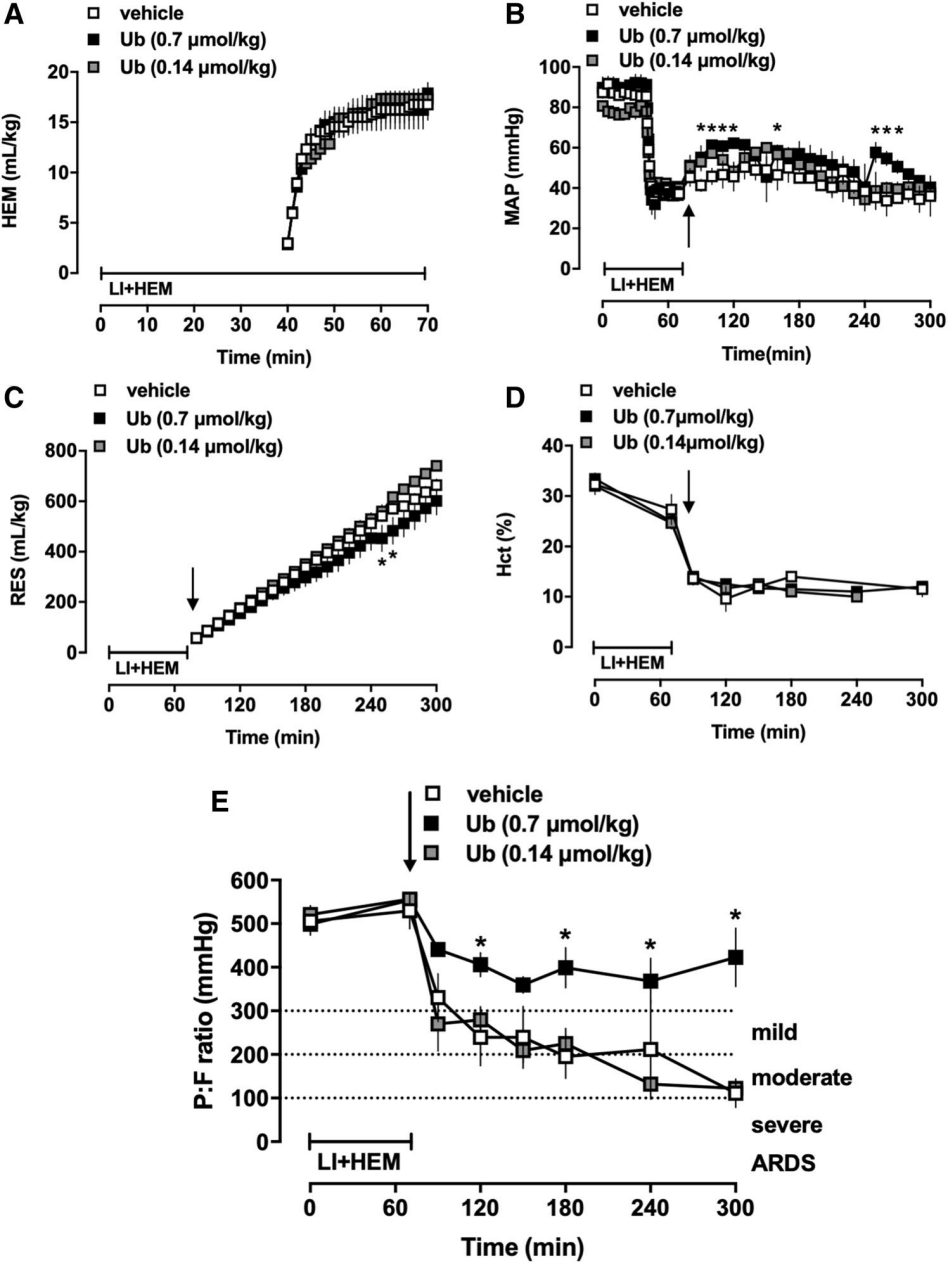
Administration of the non-cognate CXCR4 agonist ubiquitin prevents development of ARDS. Animals were treated with vehicle (n = 6), 0.7 (n = 3) or 0.14 (n = 5) μmol/kg ubiquitin. Arrows indicate the time point of drug administration. LI + HEM: Lung ischemia and hemorrhage period. Data are mean ± SE. *p < 0.05 for 0.7 μmol/kg ubiquitin vs. vehicle. (**A**) Hemorrhage volumes (mL/kg) during LI + HEM. (**B**) MAP: Mean arterial blood pressure (mmHg). (**C**) RES: Resuscitation fluid requirements (mL/kg). (**D**) Hct: Hematocrit (%). (**E**) PaO_2_/FiO_2_ (mmHg).

In the first series of experiments animals were treated with vehicle or ubiquitin (0.14 and 0.7 μmol/kg). There were no statistically significant differences in any of the measured physiological parameters between groups at baseline (Fig. 1). Hemorrhage volumes to achieve the target MAP of 40 mmHg during the shock period were comparable among the groups (Fig. 1A). Two of 6 (2/6), 2/3 and 3/5 animals after treatment with vehicle, 0.14 and 0.7 μmol/kg ubiquitin, respectively, survived until t = 300 min. MAP, resuscitation fluid requirements and hematocrit values were comparable between ubiquitin and vehicle treated animals, despite some statistically significant differences in MAP and fluid requirements during short time periods (Fig. 1B–D). Vehicle treated animals fulfilled criteria for mild ARDS between t = 90–120 min (P/F < 300 mmHg) and for moderate ARDS (P/F < 200 mmHg) at t = 180 min. The average P/F of vehicle treated animals at the end of the observation period was 110 ± 33 mmHg (Fig. 1E). While animals treated with 0.14 μmol/kg ubiquitin were indistinguishable from vehicle-treated animals, treatment with 0.7 μmol/kg ubiquitin prevented development of ARDS (Fig. 1E).

### CXCL12 treatment prevents development of ARDS

**Figure 2:**
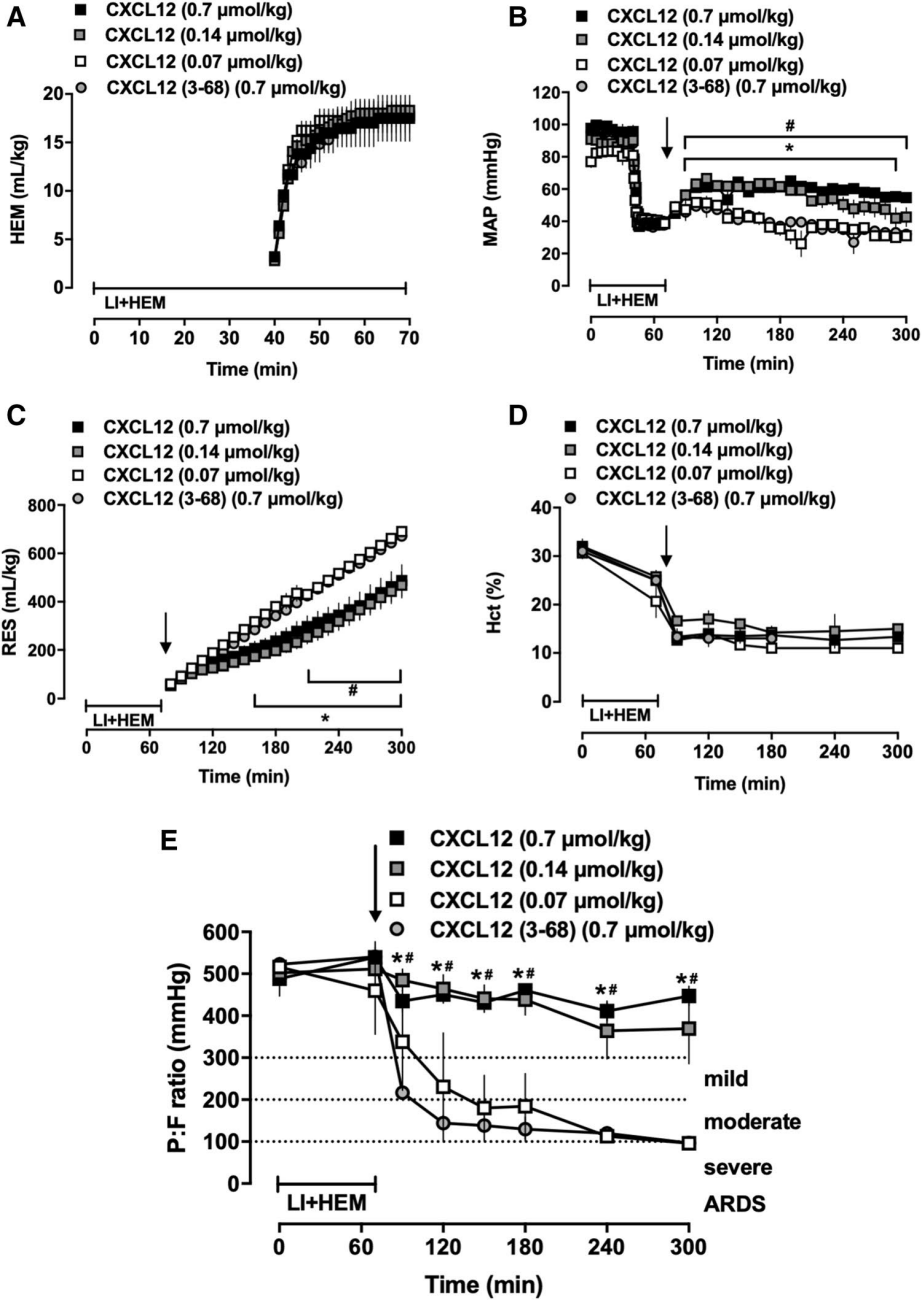
Administration of the cognate CXCR4 agonist CXCL12 prevents development of ARDS. Animals were treated with 0.7 μmol/kg inactive N-terminal truncated CXCL12 (3–68) (control, n = 3) or with 0.7 (n = 3), 0.14 (n = 5) or 0.07 (n = 3) μmol/kg CXCL12. Arrows indicate the time point of drug administration. LI + HEM: Lung ischemia and hemorrhage period. Data are mean ± SE. ^#^p < 0.05 for 0.7 μmol/kg CXCL12 vs. CXCL12 (3–68). *p < 0.05 for 0.14 μmol/kg CXCL12 vs. CXCL12 (3–68). (**A**) Hemorrhage volumes (mL/kg) during LI + HEM. (**B**) MAP: Mean arterial blood pressure (mmHg). (**C**) RES: Resuscitation fluid requirements (mL/kg). (**D**) Hct: Hematocrit (%). (**E**) PaO_2_/FiO_2_ (mmHg).

We then performed the second series of experiments, in which animals were treated with various doses of CXCL12 (0.07, 0.14 and 0.7 μmol/kg). The inactive N-terminal truncated CXCL12 (3–68) was used as a specific control protein. Animals in all groups were indistinguishable at baseline (Fig. 2). Hemorrhage volumes were comparable among the groups (Fig. 2A). One of 3 animals treated with CXCL12 (3–68) survived until t = 300 min. One of 3 (1/3), 5/5 and 3/3 animals after treatment with 0.07, 0.14 and 0.7 μmol/kg CXCL12, respectively, survived until t = 300 min. Despite similar hematocrit values, MAPs were higher and fluid requirements lower in animals treated with 0.7 μmol/kg and 0.14 μmol/kg CXCL12, as compared with animals treated with 0.7 μmol/kg CXCL12 (3–68) and 0.07 μmol/kg CXCL12 (Fig. 2B–D). Similar to vehicle treated animals in series 1, P/F in animals treated with 0.7 μmol/kg CXCL12 (3–68) and 0.07 μmol/kg CXCL12 progressively decreased and animals fulfilled criteria for moderate to severe ARDS at t = 300 min (Fig. 2E). Treatment with 0.7 μmol/kg and 0.14 μmol/kg CXCL12 prevented development of ARDS (P/F > 300 mmHg at all time points, Fig. 2E).

### Comparison of CXCL12 with engineered CXCL12 variants for prevention of ARDS

**Figure 3:**
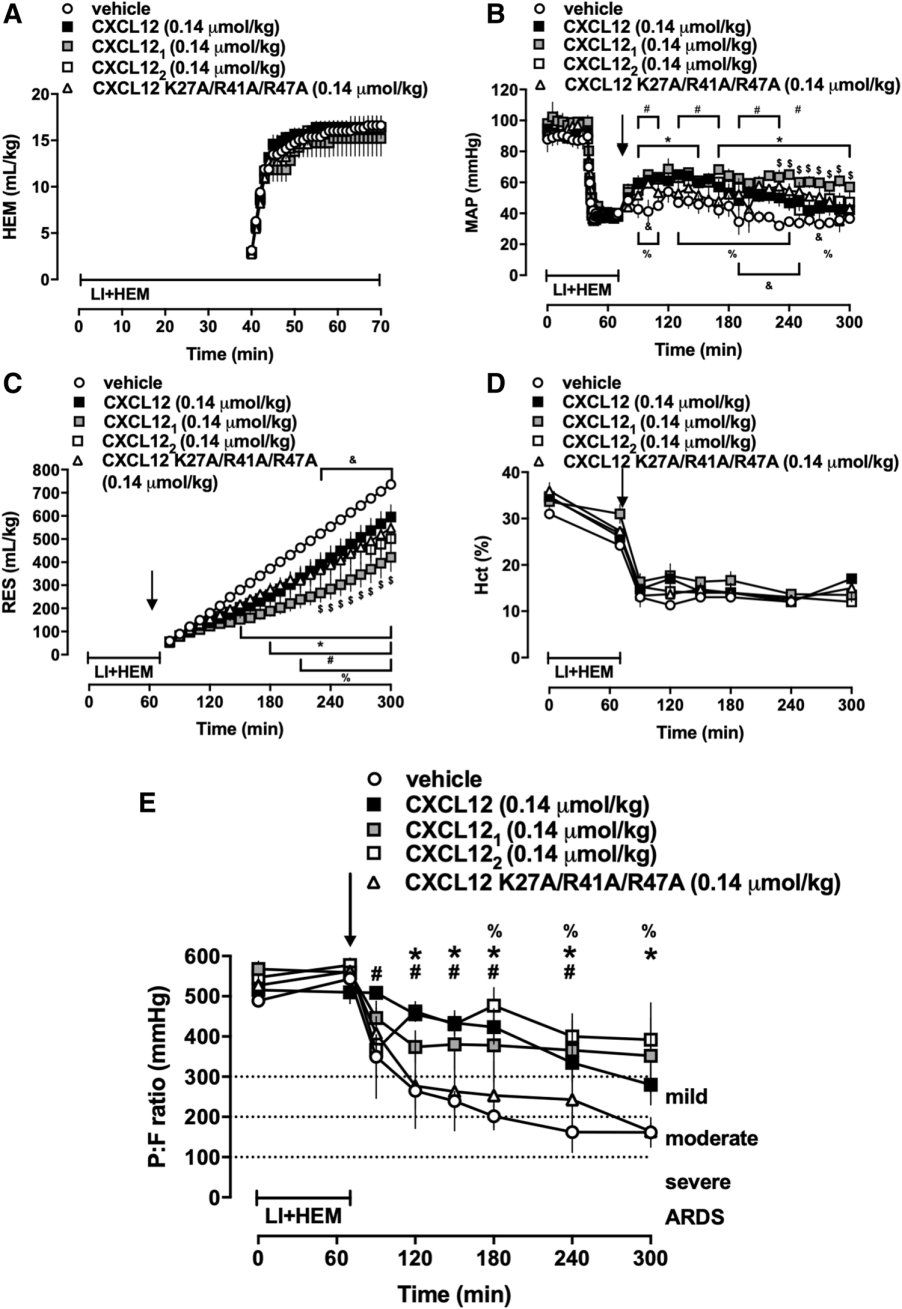
Administration of engineered CXCL12_1_ and CXCL12_2_ prevent development of ARDS. Animals were treated with vehicle (n = 5) or with 0.14 μmol/kg of CXCL12 (n = 4), CXCL12_1_ (n = 3), CXCL12_2_ (n = 4) or CXCL12 K27A/R41A/R47A (n = 3). Arrows indicate the time point of drug administration. LI + HEM: Lung ischemia and hemorrhage period. Data are mean ± SE. ^#^p < 0.05 for vehicle vs. CXCL12. *p < 0.05 for vehicle vs. CXCL12_1_. ^%^p < 0.05 for vehicle vs. CXCL12_2_. ^&^p < 0.05 for vehicle vs. CXCL12 K27A/R41A/R47A. ^$^p < 0.05 for CXCL12 vs. CXCL12_1_. (**A**) Hemorrhage volumes (mL/kg) during LI + HEM. (**B**) MAP: Mean arterial blood pressure (mmHg). (**C**) RES: Resuscitation fluid requirements (mL/kg). (**D**) Hct: Hematocrit (%). (**E**) PaO_2_/FiO_2_ (mmHg).

In the third series of experiments, we selected a dose of 0.14 μmol/kg as the lowest effective dose of CXCL12 from series 2 to compare the effects of wild-type CXCL12 with vehicle and the engineered CXCL12 variants. As in series 1 and 2, animals in all groups were indistinguishable at baseline (Fig. 3) and hemorrhage volumes to achieve the target MAP during the shock period were comparable among the groups (Fig. 3A). Three of 5 (3/5) vehicle treated animals and 4/4, 3/3, 3/4 and 2/3 animals treated with CXCL12, CXCL12_1_, CXCL12_2_ and CXCL12 K27A/R41A/R47A, respectively, survived until t = 300 min. Despite indistinguishable hematocrit values among groups, MAPs were higher and fluid requirements lower in animals treated with CXCL12 and with the CXCL12 variants, as compared with vehicle treated animals (Fig. 3B–D). Fluid requirements after CXCL12_1_ treatment were significantly lower than after treatment with CXCL12 (Fig. 3C). P/F in animals treated with vehicle fulfilled criteria for mild ARDS at t = 120 min and for moderate ARDS from t = 180 min until t = 300 min (Fig. 3E). P/F in animals treated with CXCL12 K27A/R41A/R47A were indistinguishable from vehicle-treated animals. The mean P/F of animals treated with CXCL12 fulfilled the criteria of mild ARDS not until t = 300 min. Animals treated with CXCL12_1_ and CXCL12_2_ did not fulfill criteria for ARDS at any time point (Fig. 3E).

### Treatment with CXCR4 agonists reduces mortality

**Figure 4:**
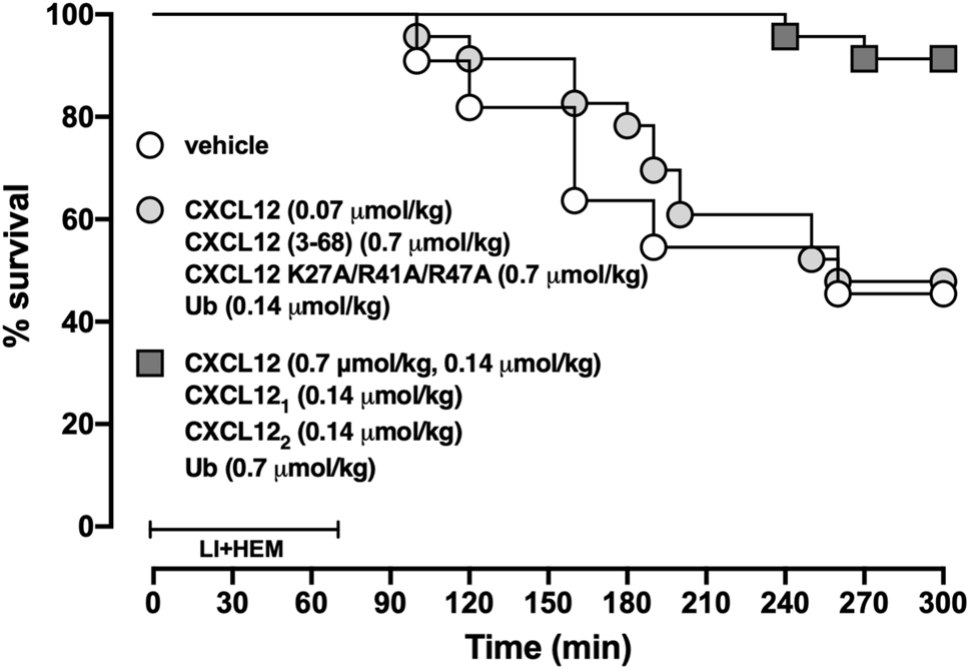
Administration of CXCR4 agonists reduce mortality from ARDS. Cumulative survival curves from animals treated with vehicle (open circles, n = 11), CXCR4 agonists administered at doses that did not prevent ARDS (grey circles, n = 12) and CXCR4 agonists administered at doses that prevented ARDS (grey squares, n = 23, p = 0.0023 vs. vehicle and CXCR4 agonists at doses that did not prevent ARDS).

Figure 4 shows the cumulative survival curves for vehicle treated animals, animals treated with inefficacious doses of the CXCR4 agonists and for animals treated with the CXCR4 agonists at doses that prevented ARDS from series 1–3. In vehicle-treated animals and animals treated with CXCR4 agonists at doses that did not attenuate ARDS development mortality was 55% and median survival time was 240 min (p > 0.05). In animals treated with CXCR4 agonists that prevented ARDS development mortality was 9% and median survival time was > 300 min (p = 0.0023 vs. vehicle-treated animals).

### Treatment with CXCR4 agonists reduces histological lung injury

**Figure 5:**
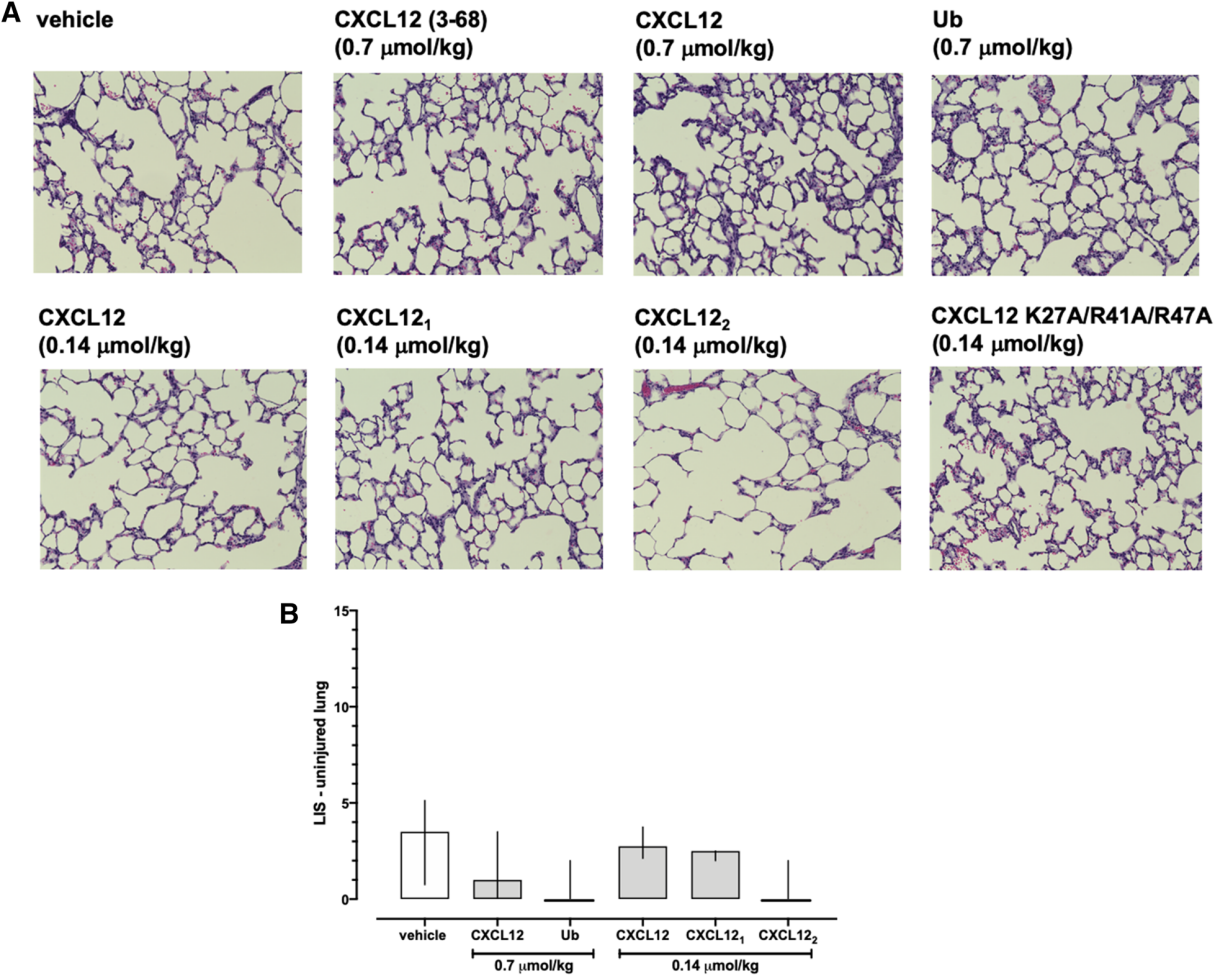
Histology of the uninjured lungs after treatment with vehicle and CXCR4 agonists. (**A**) Representative images from H&E stained lung sections from animals after treatment with vehicle and the various CXCR4 agonists. (**B**) Lung injury scores (LIS) from treatment groups in which lungs from at least 3 animals could be harvested. Data are median ± interquartile ranges. Vehicle: n = 4. 0.7 μmol/kg CXCL12: n = 3. 0.7 μmol/kg ubiquitin: n = 3. 0.14 μmol/kg CXCL12: n = 5. 0.14 μmol/kg CXCL12_1_: n = 3. 0.14 μmol/kg CXCL12_2_: n = 3. There were no statistically significant differences among groups.

**Figure 6:**
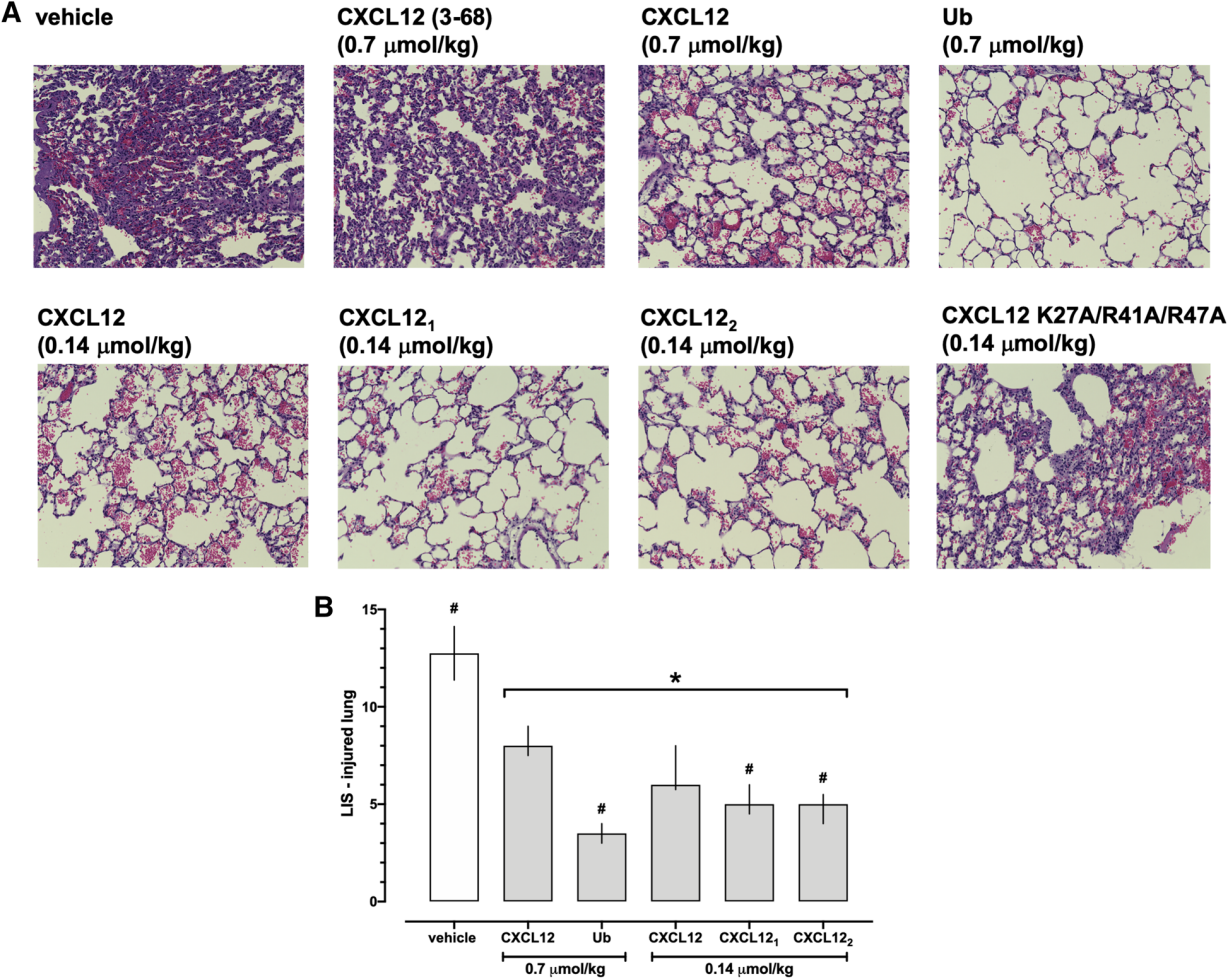
Administration of CXCR4 agonists reduce histological lung injury in the injured lung. (**A**). Representative images from H&E stained lung sections from animals after treatment with vehicle and the various CXCR4 agonists. (**B**) Lung injury scores (LIS) from treatment groups in which lungs from at least 3 animals could be harvested. Data are median ± interquartile ranges. Vehicle: n = 4. 0.7 μmol/kg CXCL12: n = 3. 0.7 μmol/kg ubiquitin: n = 3. 0.14 μmol/kg CXCL12: n = 5. 0.14 μmol/kg CXCL12_1_: n = 3. 0.14 μmol/kg CXCL12_2_: n = 3. *p < 0.05 vs. vehicle. ^#^p < 0.05 vs. 0.7 μmol/kg CXCL12.

Next, we assessed whether the effects of the CXCR4 agonists on lung function are accompanied by alterations in lung histomorphology. Representative images from H&E stained lung slices of the uninjured (left) lungs are shown in Fig. 5A. There were no obvious differences in the histomorphology of the uninjured lungs among groups. LIS in the groups in which lungs from at least three animals could be obtained at t = 300 min were not significantly different among groups (p > 0.05 vs. vehicle for all; Fig. 5B). Figure 6A shows representative images from H&E stained lung slices of the injured (right) lungs. In vehicle-treated animals and animals treated with CXCL12 (3–68) and CXCL12K27A/R41A/R47A, lung histology showed alveolar congestion, hemorrhage, interstitial edema, increased alveolar wall thickness and leukocyte infiltration, which were obviously reduced with CXCL12, CXCL12_1_, CXCL12_2_ and ubiquitin treatment (Fig. 6A). Accordingly, LIS were significantly reduced in animals after treatment with CXCR4 agonists at doses that prevented ARDS development, as compared with vehicle-treated animals (Fig. 6B). Furthermore, LIS after treatment with ubiquitin, CXCL12_1_ and CXCL12_2_ were lower than after treatment with wild-type CXCL12 (Fig. 6B).

## Discussion

In the present study, we evaluated the pharmacodynamic properties of various CXCR4 protein agonists in a rat model of lung ischemia–reperfusion injury and hemorrhage induced ARDS. There are several new findings from the present study. First, wild-type CXCL12 and the engineered variants CXCL12_1_ and CXCL12_2_ show higher potency than ubiquitin to prevent development of ARDS. Second, administration of CXCR4 agonists at doses that prevent ARDS development reduce mortality. Third, the engineered variant CXCL12_1_ shows the most favorable pharmacodynamics among the tested CXCR4 agonists.

We showed previously that in rats ventilated with FiO_2_ of 1.0, the hemorrhage protocol alone does not lead to impairment of lung function during crystalloid resuscitation within the time period studied^16^. Therefore, the thoracic injury component, i.e. thoracotomy and lung ischemia–reperfusion injury, appears to be the primary cause of ARDS in the present study. Furthermore, several model-specific procedures, such as use of isoflurane as anesthetic, continuous ventilation of the animals with FiO_2_ of 1.0 or the strict crystalloid resuscitation regimen, may constitute confounding variables that influence cardiovascular and pulmonary function^17–19^. Thus, it is obvious that our model does not fully resemble all aspects of the etiology and treatment of severely injured patients who develop ARDS. Nevertheless, and as documented by the indistinguishable physiological parameters that we measured in three different sets of control animals, the rat model that we utilized in the present study was able to induce ARDS after lung injury and hemorrhage in a highly reproducible manner.

Besides the natural CXCR4 agonists CXCL12 and ubiquitin, we selected the engineered variants CXCL12_1_, CXCL12_2_ and CXCL12 K27A/R41A/R47A for testing in the present study. These CXCL12 variants were chosen based on their efficacy and potency to protect lung endothelial cell barrier function in vitro, and based on their distinct physicochemical properties, such as resistance to proteolytic cleavage and heparan sulfate proteoglycan binding affinity, which could result in altered functional outcomes after administration of the proteins in vivo^9,20,21^.

The effects of the non-cognate CXCR4 agonist ubiquitin on ARDS development in the present study are consistent with our previous observations^2,5,6,10^. Although a synthetic CXCL12 analogue and wild-type CXCL12 have been reported to attenuate lipopolysaccharide and oleate induced lung injury, their effects on lung function, i.e. oxygenation, have not been evaluated in these previous studies^7,22^. The findings from the present study demonstrate that wild-type CXCL12, CXCL12_1_ and CXCL12_2_ are equally efficacious but more potent than ubiquitin to preserve lung function in our ARDS model. These observations are in agreement with the previously described detrimental effects of the CXCR4 antagonist AMD3100 on lung function after isolated lung ischemia–reperfusion injury, and with the lower affinity of ubiquitin for CXCR4, when compared with the affinity of CXCL12^5,23^. The observation that CXCL12 (3–68) did not affect ARDS development is consistent with the loss of function of N-terminal truncated CXCL12 in other assay systems^9,24,25^ and documents specificity of the effects of CXCL12, CXCL12_1_ and CXCL12_2_.

Although we observed previously that the CXCR4 agonist activity of CXCL12 K27A/R41A/R47A is approximately 30*-fold* lower than of wild-type CXCL12, this triple mutant protected in vitro lung endothelial barrier function with the same potency as CXCL12 and CXCL12_1_^9^. While the absence of therapeutic efficacy of CXCL12 K27A/R41A/R47A in the present study can be explained by its reduced potency to activate CXCR4, these findings also suggest that the reduced heparan sulfate binding affinity does not compensate for the reduced potency to activate CXCR4 in vivo.

In contrast to CXCL12 and the engineered CXCL12 variants, ubiquitin binding to CXCR4 does not induce β-arrestin recruitment to recombinant CXCR4 and ubiquitin does not bind to atypical chemokine receptor 3 (ACKR3)^9,23,26–28^. Because CXCL12 K27A/R41A/R47A activates ACKR3 with high potency similar to wild-type CXCL12^9^, our findings imply that activation of ACKR3 does not contribute to the lung protective properties of CXCL12 and the CXCL12 variants. Therefore, our observations support the concept that natural and engineered CXCR4 agonists could be used as lung protective therapeutics for ARDS. Based on the significant effects of the CXCR4 agonists on survival in the present study, such a treatment may have the potential to reduce mortality from ARDS. The majority of animals who died, however, had P/F ratios above 100 mmHg and hematocrit values were indistinguishable among groups. While these observations make hypoxemia as well as fluid overload with subsequent right heart failure as causes of mortality unlikely, circulatory failure during fluid resuscitation could explain mortality in our model. These findings resemble the clinical observations that refractory hypoxemia is a rare cause of death in ARDS patients and that mortality is predominantly caused by multiple organ failure, with cardiovascular and acute renal failure being the most common organ failures^29^.

It is known that CXCL12 exists at a physiological pH in a monomer–dimer equilibrium^30^. While monomeric CXCL12 exists at low concentrations, dimerization occurs at high concentrations and is influenced by pH, phosphate, sulfate or heparin^20,31^. Although wild type CXCL12, CXCL12_1_ and CXCL12_2_ showed comparable CXCR4 and ACKR3 activities in Presto-Tango β-arrestin recruitment assays, distinct pharmacokinetic and pharmacodynamic properties have been observed in various other signaling and cell function assays in vitro, in an isolated rat heart ischemia–reperfusion injury model and in a melanoma lung metastasis model^21,32,33^. The direct side-by-side comparison of wild-type CXCL12, CXCL12_1_ and CXCL12_2_ in the present study suggests that CXCL12_1_ is the protein therapeutic with the most favorable pharmacodynamic properties when effects on lung function, lung histomorphology and hemodynamics are considered comprehensively.

While the present study was not designed to address the molecular mechanisms by which CXCR4 activation prevents ARDS development, several lines of evidence suggest that CXCR4 activation protects endothelial barrier function, and regulates vascular smooth muscle function and blood pressure through heteromerization with α_1_-adrenergic receptors^9,11,34–37^. Thus, direct protective effects of CXCR4 agonists on lung endothelial barrier function as well as improvement of cardiovascular function with subsequently reduced fluid resuscitation requirements upon CXCR4 activation likely contributed to the beneficial effects that we observed in the present study. CXCR4 and ACKR3, however, are known to hetero-oligomerize with each other and with several other G protein coupled receptors, which likely affects their signaling properties depending on the available repertoire of interacting receptor partners^34,35,38–40^. Furthermore, the natural CXCR4 agonists CXCL12 and ubiquitin are proteolytically processed in the systemic circulation, leading to their inactivation or the generation of CXCR4 antagonists, which will result in altered ligand binding kinetics at the receptor and modulate subsequent signaling events^24,41,42^. These complex mechanisms likely correspond to the observations that signaling and functions of CXCR4 and its agonists depend on the cellular context and the specific pathophysiological environment^34,38^. Thus, context-dependent consequences of CXCR4 activation with CXCL12 may explain that CXCL12 administration desensitized phenylephrine-mediated vasoconstriction of isolated arteries and reduced blood pressure in a model of isolated hemorrhagic shock in spontaneously breathing rats, whereas CXCL12 sensitized the phenylephrine induced blood pressure response in normal rats and stabilized hemodynamics in the present ARDS model^34,37^.

In conclusion, our findings suggest that the class of CXCR4 protein agonists prevent development of ARDS and reduce mortality in rats after lung ischemia–reperfusion injury and hemorrhage when administered during the early resuscitation period. The observation that engineered CXCL12_1_ outperformed ubiquitin and wild-type CXCL12 in its overall pharmacodynamic properties implies that the development of new engineered protein therapeutics with improved pharmacological properties for ARDS is possible. While the context-dependency of CXCL12 mediated effects restricts translational potential, engineered protein therapeutics may overcome this limitation. Although our study demonstrates that administration of CXCR4 agonists prevents ARDS development when administered within a few minutes upon initiation of crystalloid resuscitation, the therapeutic window for such an intervention as well as the possible therapeutic potential to improve lung function and to reduce lung injury in animals that already fulfill criteria for ARDS remain to be determined in the future. The findings from the present study provide justification for such translational pre-clinical studies.

## Methods

### Proteins

Ubiquitin, CXCL12, the engineered constitutively monomeric (CXCL12_1_) and dimeric (CXCL12_2_) CXCL12 variants, N-terminal truncated CXCL12 (3–68), a posttranslational modification that occurs in vivo after cleavage of CXCL12 by CD26/dipeptidyl peptidase 4 and inactivates the chemokine, and the triple mutant CXCL12 K27A/R41A/R47A, which shows significantly reduced heparan sulfate proteoglycan binding properties, were as described^9^.

### ARDS model

All procedures were performed according to National Institutes of Health Guidelines for Use of Laboratory Animals and were approved by the Institutional Animal Care and Use Committee and the Animal Care and Use Review Office of the U.S. Army Medical Research and Materiel Command. Male Sprague–Dawley rats (300–350 g) were purchased from Harlan (Indianapolis, IN, USA). Anesthetized (isoflurane inhalation) animals were oro-tracheally intubated with a 16-gauge EXEL disposable safelet angiocatheter (EXELINT International, Los Angeles, CA, USA) and mechanically ventilated with a SomnoSuite small animal anesthesia system (Kent Scientific Corporation, Torrington, CT, USA). Animals were ventilated with a pressure-controlled ventilator mode with an initial positive end expiratory pressure (PEEP) of 2 mmHg, a fraction of inspired oxygen (FiO_2_) of 1.0 and anesthetized with isoflurane at 2.5%. Tidal volumes were set based on the weight of the animals then titrated to maintain normal PaCO_2_ (35–45 mmHg). The femoral artery was then cannulated with 24-gauge BD angiocath shielded IV catheters (Becton, Dickinson and Company, Franklin Lakes, NJ, USA) to allow for monitoring of arterial blood pressure and blood withdrawal, and the femoral vein was cannulated with 1.5-french tubing for fluid and drug administration. Animals underwent a right lateral thoracotomy and a suture was placed around the hilum of the right lung. The suture was then tied around the hilum of the right lung, occluding the pulmonary artery, vein, and right main stem bronchus (t = 0 min). After 40 min of lung ischemia animals were hemorrhaged to a mean arterial blood pressure (MAP) of 40 mmHg until t = 70 min. At t = 70 min the suture was removed, animals were ventilated with FiO_2_ 1.0, PEEP 5 mmHg and resuscitated to a MAP of 60 mmHg with normal saline (NS). After initial bolus injections of NS to achieve the MAP target, NS infusion was limited to maximal infusion rates (2 mL/min until t = 75 min; 1 mL/min from t = 75–300 min) irrespective of the MAP target to prevent acute fluid overload. A sigh breath was administered every 15 breaths for the first 5 min following resuscitation and then every 90 breaths until t = 300 min to fully expand the post-ischemic lung. Hemodynamics were continuously monitored with the surgivet invasive blood pressure monitor (Med-Electronics, Beltsville, MD, USA) and blood pressures values were recorded every 5 min throughout the experiment. Arterial blood gases and routine laboratory parameters were determined in regular intervals throughout the experiment. At t = 300 min, animals were euthanized (5% isoflurane, bilateral pneumothorax, arterial exsanguination). Both lungs were then harvested for the analysis of lung histopathology. All experiments were performed randomized and blinded. All drugs were administered in 0.5 mL NS (= vehicle) within 5 min of fluid resuscitation. In series 1, animals received vehicle (n = 6), 0.7 μmol/kg ubiquitin (n = 3) or 0.14 μmol/kg ubiquitin (n = 5). In series 2, animals were treated with 0.7 (n = 3), 0.14 (n = 5) or 0.07 (n = 3) μmol/kg CXCL12 or with CXCL12 (3–68) (0.7 μmol/kg, n = 3). In series 3 animals were treated with vehicle (n = 5) or with 0.14 μmol/kg of CXCL12 (n = 4) or the engineered variants CXCL12_1_ (n = 3), CXCL12_2_ (n = 4) and CXCL12 K27A/R41A/R47A (n = 3). The dosing of the CXCR4 agonists was selected based on our previous observations in other animal models^5,10,43^.

### Arterial blood gases and routine laboratory parameters

Arterial blood gases, electrolytes, creatinine, lactate, hematocrit and hemoglobin were analyzed using the Element point of care veterinary blood gas, electrolyte and critical care analyzer (Cuattro Veterinary USA, Loveland, CO, USA). Complete blood counts were analyzed using the Hematrue hematology analyzer (Cuattro Veterinary USA, Loveland, CO, USA).

### Histopathology

For histomorphological examination, lung specimens were placed in formalin fixative, embedded in paraffin wax, sliced into 5 µm sections and stained with hematoxylin and eosin (H&E). From each lung specimen, 3 slides were prepared. The slides were examined under a light microscope by 4 investigators who were blinded as to the identity of the specimens. Histopathology was assessed using a previously described lung injury score (LIS)^5,44^. In brief, each investigator rendered a score of 0 (no damage) to 4 (maximal damage) based on the following criteria: (1) alveolar congestion, (2) presence of hemorrhage, (3) interstitial edema, (4) alveolar wall thickness, (5) infiltration of polymorphonuclear neutrophils (PMN) in the vessel wall and (6) infiltration of PMN in the alveoli. For each criterion the median of the scores from each investigator was used as the final score for each criterion and the sum of the scores for all six criteria calculated for each animal.

### Data analyses and statistics

Data are presented as mean ± standard error (SE) or median with interquartile range (25th/75th percentile). Data were analyzed by 1-way ANOVA or 2-way ANOVA with Dunnett's multiple comparisons test, as appropriate. Survival curves were analyzed using the log-rank test. Data analyses were calculated with the GraphPad Prism program (GraphPad Software). A two-tailed p < 0.05 was considered significant.

### Conference presentation

Parts of this research have been presented at the 42nd Conference on Shock, Coronado, CA, 2019 (Shock, 51(S1):36, 2019).

## Data Availability

The datasets generated during the current study are available from the corresponding author on reasonable request.
